# Retrospective Seroepidemiology study of dengue virus infection in Taiwan

**DOI:** 10.1186/s12879-021-05809-1

**Published:** 2021-01-21

**Authors:** Ying-Hsuan Lee, Yu-Chia Hsieh, Chih-Jung Chen, Tzou-Yien Lin, Yhu-Chering Huang

**Affiliations:** 1 Chang Gung University College of Medicine, Gueishan, Taoyuan, Taiwan; 2 Department of Pediatrics, Division of Pediatric Infectious Diseases, Chang Gung Memorial Hospital, No. 5, Fu-Shin Street, Gueishan, 333 Taoyuan, Taiwan

**Keywords:** Seroepidemiology, Dengue fever, Taiwan, Nationality

## Abstract

**Background:**

Dengue virus infection has been an important and serious public health concern in Taiwan, where local outbreaks of dengue fever occurred almost every year. To our knowledge, no nationwide investigation has been carried out to determine the actual extent of infection in the general population.

**Methods:**

A total of 1308 random serum samples were collected from the general population in Taiwan in 2010. The antibody-captured enzyme-linked immunosorbent assays were used to detect DENV-specific IgM and IgG. Demographics data were used for risk analysis.

**Results:**

The weighted overall seroprevalence was 1.96% for anti-DENV IgM, and 3.4% for anti-DENV IgG, respectively. A significant rise of DENV IgG seropositive rate had been noted since late adulthood stage, from 1.1% at the age group of 50–59 years to 7.6% at the age group of 60–69 years. For people aged over 70 years, the seropositive rate reached 19%. Age, nationality, and regions of residency were associated with the IgG seropositivity. There was no statistically significant difference in seroprevalence of anti-Dengue IgM, indicating recent infection, among univariate predictors we proposed, including gender, age, residency, nationality, and household size.

**Conclusions:**

Our results indicated that the majority of population in Taiwan born after 1940 is naive to dengue virus and the prevalence of IgG antibody against dengue virus rises with age. Nationality, and regions of residency are associated with the exposure of population to infection by dengue viruses. Further studies are needed to realize the current situation of seroprevalence of dengue fever in Taiwan.

## Introduction

Dengue infection is a major global burden caused by four types of dengue viruses (DENV-1, DENV-2, DENV-3, DENV-4) belonging to *Flaviviridae* family. The viruses are transmitted through the bite of infected *Aedes aegypti* and *Aedes albopictus* female mosquitoes. Recovery from one episode of infection provides lifelong immunity against that particular serotype, yet the cross-immunity to the other serotypes is temporary and partial [1, 2]. There is no specific treatment for dengue infections.

Clinical manifestations of dengue virus infection range from asymptomatic, mild flu-like symptoms, to severe life-threatening dengue complications such as dengue shock syndrome (DSS) and dengue hemorrhagic fever (DHF) [3]. DHF/DSS cases are associated with a secondary-type dengue antibody response, which makes the second dengue infection worse than the first due to “antibody-dependent enhancement of infection” [3]. Those asymptomatic infection cases, which induce an antibody response but lacks clinical symptoms requiring a medical consultation, pose challenges to disease prevention programs.

The incidence of dengue has grown dramatically worldwide in recent years. Recent studies estimated that 500,000 people with severe dengue require hospitalization each year, and about 2.5% of those affected die [4, 5]. Besides the growing incidence globally, the hyper-endemicity of multiple dengue virus serotypes in many regions also pose an alarming impact on public heath as well as national economies.

According to the previous study, Taiwan is not yet a dengue endemic region. Local outbreaks of dengue fever, however, occurred almost every year, which is believed to result from the constant importation of dengue infections from neighboring Southeast Asian countries [6]. The majority of outbreaks were reported in southern Taiwan, but occasionally in northern Taiwan [1, 7]. Notably, two consecutive severe dengue epidemics occurred in 2014 and 2015 in southern Taiwan [8]. The death rate in 2015 DHF/DSS cases was higher than previous dengue epidemics or outbreaks.

In recent years, arboviruses that cause dengue, chikungunya, and Zika illnesses have rapidly expanded across the globe. The more recent Zika outbreaks have caused severe neurological complications including Guillain–Barré Syndrome and birth defects, of which the immunopathogenesis is enhanced in the setting of high seroprevalence of dengue antibodies due to structural similarities between Zika and dengue virus [9, 10]. It is important to study dengue virus seroprevalence to project future epidemic patterns in Taiwan. There have been scanty studies of seroprevalence of dengue virus from Taiwan, all from the southern part of Taiwan but not the whole island, and few results were published in the literature. Considering the massive population morbidity and the high rates of asymptomatic cases, existing studies were not enough for determining the current immune status of dengue in the community [11–13]. This pilot study was the first study to investigate the seroprevalence, measured by the presence of IgG and IgM antibodies of dengue virus in general populations from different geographical areas in Taiwan to figure out the whole picture of dengue disease prevalence in Taiwan.

## Materials and methods

### Study samples

In this study, 1308 human sera were selected from samples collected for one survey in 2010 [14]. This survey was conducted to investigate the seroprevalence of the pandemic influenza A H1N1 virus in Taiwan and a total of 1558 samples were obtained between September and October of 2010. Briefly, the survey used a multi-stratified design to sample the civilians from three regions (northern part of Taiwan: Taipei, Taoyuan; and southern part of Taiwan: Tainan). In each region, age- and gender-stratified sampling was conducted using household registration records. A questionnaire-based interview was used to collect demographic data at the time of blood sampling. Information about travel history (history of traveling to an endemic area), movement history, types of dwelling. Etc., was not collected in the serosurvey in 2010 and therefore not analyzed in the study.

To examine the DENV seroprevalence in the Taiwanese population, we selected residual samples from all age groups. Age was grouped into eight categories: 0–9, 10–19, 20–29, 30–39, 40–49, 50–59, 60–69, and ≧70 years. The serum samples were stored at − 80 °C.

#### Ethical review

The Institute Review Board of Chang Gung Memorial Hospital approved this study and all subjects provided written informed consent.

### Laboratory methods

The DENV-specific IgM and IgG of the samples were determined by 2 commercial antibody-captured enzyme - linked immunosorbent assay (ELISA) systems (EUROIMMUN, Germany). The sensitivity and specificity of the IgM ELISA kit were 100 and 98%, respectively, with a cut-off ratio of 1.0 relative units (RU)/ml. As for the IgG ELISA kit, the test showed a specificity of 100% and a sensitivity of 100%, with a cut-off ratio of 1.1 relative units (RU)/ml. In addition, both the intra- and inter-assay coefficients of variability were less than 10% for anti-DENV IgM and anti-DENV IgG kits, respectively.

The analysis of anti-DENV responses was performed following the manufacturer’s instructions. In short, the serum was diluted 1:100 and loaded to the pre-coated microplates. After incubation at room temperature for 30 min, the plates were washed and incubated with peroxidase-labelled anti-human IgM/ IgG at room temperature for 30 min. Following the further wash, the plates were incubated with kit substrates at room temperature for 15 min. Positive controls, negative controls, and calibration standards were included on each plate. Photometric measurement of the color intensity was made at a wavelength of 450 nm. The standard curve was established by calibrators and used to calculate the antibody concentration.

According to the previous reviews, antibodies of class IgM are detectable from the 5th day of illness and for 3–6 months following initial infection [15, 16]. IgM antibodies are often not detectable after a second infection with another serotype [17]. Antibodies of class IgG arise several days later than IgM and probably persist for life. Therefore, individuals found positive for IgG were classified as those with past dengue virus infection, while those found positive for IgM were classified as recent dengue virus infection.

### Statistics

The data were analyzed with the SPSS Statistics, version 26.0 (2019 SPSS Inc., Chicago, IL, USA). In the interest of representativity as regard to the general population, each participant was weighted by post stratification according to age, gender in order to account for unequal probabilities of selection. In weighting, the structure of the total population in Taipei, Taoyuan, and Tainan from 2010 census data was used as the reference (available at https://www.ris.gov.tw/346). Weighted seroprevalence data are reported as percentages (%) with a 95% confidence interval. Comparison of population profile in general population and subjects recruited in this study was illustrated in Table 1. Regarding missing data for certain categories, percentages were determined by excluding those with missing data from the denominator.

**Table 1:** Comparison of population profile in Taiwan and subjects recruited in the study

Characteristics	Subjects recruited in this study		^a^2010 population by census	
	Number	%	Number	%
Gender				
Male	517	39.5	5,151,540	49.6
Female	791	60.5	5,240,453	50.4
Age groups (years)				
0–9	246	18.8	944,019	9.1
10–19	154	11.8	1,371,595	13.2
20–29	180	13.8	1,542,079	14.8
30–39	196	15	1,747,486	16.8
40–49	178	13.6	1,728,634	16.6
50–59	177	13.5	1,555,250	15.0
60–69	119	9.1	780,058	7.5
≥ 70	58	4.4	722,872	7.0
Locality				
Taipei	494	37.8	6,516,139	62.7
Taoyuan	489	37.4	2,002,060	19.3
Tainan	325	24.8	1,873,794	18.0

^a^Total population of Taipei, Taoyuan, and Tainan form 2010 census data

Individual univariate qualitative variables were first analyzed for association with dengue seropositivity. Chi-squared test was used to test for statistical significance in categorical variables as appropriate. Spearman’s correlation coefficient was used to examine the strength of relationship between the age and the seropositive rate. An analysis of variance (ANOVA) used for comparisons of IgM/ IgG between cities. Variables with a *P* value less than 0.20 were entered into the multivariable logistic regression model and odds ratios along with 95% CI were reported [18]. All *P* values reported were two- sided and statistical significance was taken at *P* <  0.05.

## Results

### Demographic profile of participants

The demographic characteristics of the participants are shown in Table 1. The 1308 serum samples were obtained from 791 (60.5%) females and 517 (39.5%) males. The mean age of the participants was 42.4 years (range 0–92 years, standard deviation 21.4). Among them, 983 subjects (75.2%) and 325 (24.8%) were from northern district (Taipei and Taoyuan) and southern district (Tainan), respectively.

### Seropositivity of anti-DENV IgM among participants

Of the 1308 study subjects, the overall weighted seropositive rate of anti-DENV IgM was 1.76% (95%CI, 1.74–1.78, Table 2) Weighted seropositive rates among females and males were 2.47% (95% CI, 2.42–2.52%) and 1.21% (95% CI, 1.18–1.24%), respectively. The incidence of IgM antibodies was highest in the age group of 0–9 years (3.70, 95% CI, 3.39–4.01%) followed by the age group of those 70 years or above (3.47, 95% CI, 3.08–3.81%); seroprevalence fluctuated between 0.61 ~ 2.13% for participants aged 10 ~ 69 years. No statistical relationship was observed between IgM-positive subjects and age groups (*p* = 0.658).

**Table 2:** Univariate predictors of dengue IgM seropositivity in Taiwan

Characteristics	No. positive/ No. tested	Weighted prevalence (%)	(95% CI)	Odds Ratio	95% CI	*P value
Gender						0.009
Male	10/517	1.21		Referent		
Female	18/791	2.47		2.071	(0.877, 4.889)	
Age groups (years)						0.658
0–9	10/246	3.70	(3.39, 4.01)	Referent		
10–19	0/154	0.61	(0.52, 0.69)	0.159	(0.019, 1.356)	
20–29	2/180	1.36	(1.25, 1.48)	0.36	(0.077, 1.683)	
30–39	4/196	1.57	(1.46, 1.68)	0.414	(0.099, 1.728)	
40–49	5/178	1.56	(1.44, 1.67)	0.411	(0.098, 1.728)	
50–59	3/177	2.13	(1.98, 2.27)	0.566	(0.145, 2.410)	
60–69	2/119	1.92	(1.65, 2.20)	0.51	(0.091, 2.870)	
≥ 70	2/58	3.47	(3.08, 3.87)	0.936	(0.215, 4.081)	
Total		1.76	(1.74, 1.78)			
Locality						0.766
Taipei	9/ 494	1.75		Referent		
Taoyuan	12/ 489	2.18		1.252	(0.506, 3.097)	
Tainan	7/325	1.50		0.855	(0.282, 2.590)	
Nationality						0.582
Taiwanese	27/1227	1.83				
Immigrants	0/21	0				
Household size						0.455
1–4	13/648	1.66		Referent		
≥ 5	10/456	2.28		1.402	(0.591, 3.33)	

*CI* Confidence interval. Prevalence, Odds Ratio, and CIs are based on weighted data

Missing data: Nationality (*n* = 60), Household size (*n* = 205). Percentages determined by excluding those with missing data from the denominator

One sample collected from one 51-year-old male, who lived in Taipei with a household size of four, showed positive for both IgM and IgG

Participants in Taoyuan had the highest weighted seroprevalence of anti-DENV IgM (2.18, 95% CI, 2.12–2.25%), followed by Taipei (1.75, 95% CI, 1.70–1.80%) and Tainan (1.50, 95% CI, 1.43–1.57%). DENV IgM was not detected in any of the sera samples collected from immigrants, while the seroprevalence of DENV IgM in Taiwanese participants was 1.83%.

Based on univariate analysis, the relationship between DENV IgM and the other sociodemographic characteristics (age, region of residence, nationality, or household size) did not show any statistically significant association (Table 2).

### Seropositivity of anti-DENV IgG among participants

The overall weighted seropositive rate of anti-DENV IgG was 3.40% (95% CI, 3.37–3.42%). The weighted seropositive rates among women and men were 3.52% (95% CI, 3.47–3.58%), and 3.27% (95% CI, 3.21–3.32%) respectively. No significant difference in seropositivity of IgG between male and female participants was detected (*p* = 0.796).

According to the univariate analysis, statistically significant differences in seropositivity of IgG were found between age group (*p* <  0.0001) (Table 3). A significant rise of DENV IgG seropositive rate had been noted among those ages 50 and over, from 1.23% at the age group of 50–59 years to 8.33% at the age group of 60–69 years. For people aged 70 years and above, the seropositive rate reached 18.25%. In addition, the nationality (IgG seropositive rate among immigrants: 28.82%, *p* <  0.0001) was associated with the DENV IgG seropositivity. Region of residence was statistically associated with seropositive rates of DENV IgG (*p* = 0.001); the prevalence of dengue IgG positives were highest in subjects residing in Tainan with 6.64%, followed by 2.76% in Taipei and 1.81% in Taoyuan. No statistical relationship was observed between IgG-positive subjects and household size (*p* = 0.302).

**Table 3:** Univariate analysis of dengue IgG seropositivity in Taiwan

Characteristics	No. positive/ No. tested	Weighted prevalence (%)		Odds Ratio	95% CI	*P value
Gender						0.796
Male	18/517	3.27		Referent		
Female	266/791	3.52		1.082	(0.594, 1.971)	
Age groups (years)						< 0.0001
0–9	6/246	2.41	(2.15, 2.67)	Referent		
10–19	2/154	1.21	(1.09, 1.33)	0.498	(0.082, 3.004)	
20–29	3/180	1.62	(1.49, 1.74)	0.666	(0.132, 3.36)	
30–39	9/196	3.38	(3.23, 3.54)	1.419	(0.356, 5.652)	
40–49	2/178	0.78	(0.69, 0.86)	0.318	(0.047, 2.154)	
50–59	2/177	1.23	(0.13, 1.33)	0.504	(0.089, 2.842)	
60–69	9/119	8.33	(7.79, 8.88)	3.684	(0.932, 14.555)	
≥ 70	11/58	18.25	(17.41,19.09)	9.046	(2.496, 32.793)	
Total		3.40	(2.30, 4.49)			
Locality						0.001
Taipei	15/ 494	2.76		Referent		
Taoyuan	11/ 489	1.81		0.651	(0.275, 1.537)	
Tainan	18/325	6.64		2.504	(1.262, 4.969)	
Nationality						< 0.0001
Taiwanese	36/1227	3.06		Referent		
Immigrants	6/21	28.82		12.821	(4.180, 39.325)	
Household size						0.302
1–4	17/648	2.67		Referent		
≥ 5	16/455	3.76		1.424	(0.725, 2.795)	

*CI* Confidence interval. Prevalence, Odds Ratio, and CIs are based on weighted data

Missing data: Nationality (*n* = 60), Household size (*n* = 205). Percentages determined by excluding those with missing data from the denominator

One sample collected from one 51-year-old male, who lived in Taipei with a household size of four, showed positive for both IgM and IgG

In the multivariate logistic regression model, individuals aged 60 or older, immigrant and individuals living in Tainan were found to be independent risk factors significantly associated with IgG seroposivity (Table 4).

**Table 4:** Multivariate logistic regression model of risk factors associated with dengue IgG seropositivity in Taiwan

Characteristics	No. positive/ No. tested	Adjusted OR	95% CI	*P* value
Nationality				< 0.0001
Taiwanese	36/1227	Referent		
Immigrants	6/21	51.887	(12.766, 210.898)	< 0.0001
Age groups (years)				< 0.0001
0–9	6/246	(referent)	–	
10–19	2/154	0.574	(0.084, 3.919)	0.571
20–29	3/180	0.840	(0.148, 4.754)	0.844
30–39	9/196	1.452	(0.315, 6.698)	0.632
40–49	2/178	0.299	(0.039, 2.294)	0.246
50–59	2/177	0.261	(0.022, 3.118)	0.289
60–69	9/119	4.776	(1.045, 21.839)	0.044
≥ 70	11/58	13.283	(3.144, 56.112)	< 0.0001
Locality				< 0.001
Taipei	15/ 494	(referent)		
Taoyuan	11/ 489	0.526	(0.188, 1.467)	0.220
Tainan	18/325	3.262	(1.518, 7.007)	0.000

*CI* Confidence interval. Adjusted OR, and CIs are based on weighted data

Missing data: Nationality (*n* = 60). Percentages determined by excluding those with missing data from the denominator

### The joint seropositivity of antibodies (Ab) IgM and IgG

The entire weighted seroprevalence of dengue-specific antibodies (IgM and/or IgG) in the population studied was 6.78%. Among the study subjects, only one sample showed seropositive to both IgM and IgG.

### Comparison of seroprevalence between regions

**Fig. 1:**
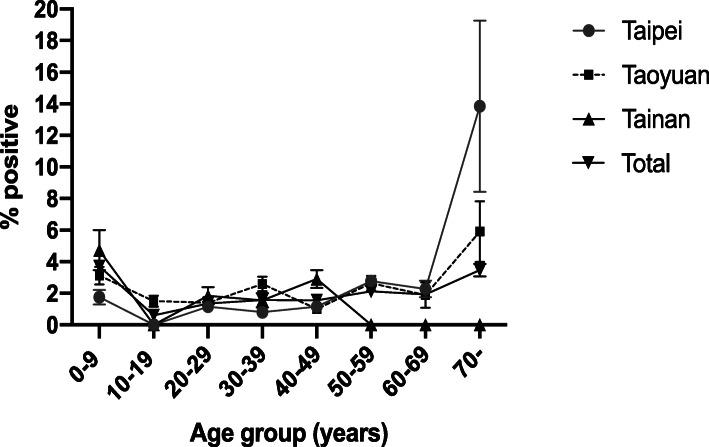
Age group-specific anti-DENV IgM seroprevalence by region

The seroprevalence of antibodies against dengue virus for each region, by age and gender, is summarized (Tables 5 and 6). As previously mentioned above, the overall seroprevalence of anti-DENV IgM was highest in Taoyuan (2.18, 95% CI, 2.12–2.25%), followed by Taipei (1.75, 95% CI, 1.70–1.80%) and Tainan (1.50, 95% CI, 1.43–1.57%). Among individuals 0–9 years of age, the highest prevalence was in Tainan (4.73%). The prevalence of anti-dengue IgM was then declined and remained relatively low among individuals 10–69 years of age regardless of the region in which they resided (0% ~ 2.78%) (Fig. 1). The seroprevalence sharply increased for individual aged 70 years and above in Taipei and Taoyuan, reaching 13.84 and 5.90%, respectively. The shape of the curve of seropositivity by age between Taipei and Taoyuan was very similar. In Tainan, the seroprevalence was highest among individuals 0–9 years of age (4.73%). No IgM seropositive case was detected in the 10–19-year-old, 50-year-old and over age group in Tainan.

**Table 5:** Seroprevalence of anti-dengue virus IgM and anti-dengue virus IgG stratified by age groups and cities^a^

Characteristics		Taipei		Taoyuan		Tainan	
		Seroprevalence (%)	95% CI	Seroprevalence (%)	95% CI	Seroprevalence (%)	95% CI
Age groups (years)							
0–9	IgM	1.76	(1.29, 2.22)	3.12	(2.55, 3.68)	4.73	(3.45, 6.01)
	IgG	0.93	(0.59, 1.26)	3.03	(2.76, 3.30)	1.58	(0.82, 2.33)
10–19	IgM	0.00	(0.00, 0.00)	1.50	(1.16, 1.84)	0.00	(0.00, 0.00)
	IgG	0.00	(0.00, 0.00)	0.00	(0.00, 0.00)	4.48	(3.61, 5.35)
20–29	IgM	1.15	(0.88, 1.42)	1.40	(1.04, 1.77)	1.84	(1.29, 2.39)
	IgG	0.00	(0.00, 0.00)	1.40	(1.02, 1.79)	4.69	(3.83, 5.56)
30–39	IgM	0.81	(0.64, 0.99)	2.59	(2.14, 3.05)	1.55	(1.10, 2.01)
	IgG	4.07	(3.69, 4.45)	2.40	(1.92, 2.87)	3.11	(2.47, 3.75)
40–49	IgM	1.16	(0.87, 1.45)	1.01	(0.78, 1.25)	2.90	(2.34, 3.47)
	IgG	1.16	(0.87, 1.45)	1.01	(0.75, 1.27)	0.00	(0.00, 0.00)
50–59	IgM	2.78	(2.41, 3.16)	2.64	(2.17, 3.11)	0.00	(0.00, 0.00)
	IgG	2.78	(2.41, 3.16)	0.00	(0.00, 0.00)	0.00	(0.00, 0.00)
60–69	IgM	2.27	(1.75, 2.80)	1.89	(1.09, 2.69)	0.00	(0.00, 0.00)
	IgG	3.41	(2.77, 4.06)	5.66	(4.59, 6.74)	18.92	(15.62, 22.22)
≥ 70	IgM	13.84	(8.43, 19.25)	5.90	(3.99, 7.82)	0.00	(0.00, 0.00)
	IgG	34.1	(26.74, 41.58)	5.90	(2.84, 8.97)	41.31	(37.66, 44.97)
Total	IgM	1.75	(1.70, 1.80)	2.18	(2.12, 2.25)	1.50	(1.43, 1.57)
	IgG	2.76%	(2.70, 2.83)	1.81%	(1.77, 1.86)	6.64%	(6.49, 6.79)

*CI* Confidence interval

^a^All data were weighted

**Table 6:** According to a database of dengue cases covering the period 1998 ~ 2010, 8956 of 14,013(63.9%) confirmed dengue cases were reported from Kaohsiung

Region	Dengue Cases
Taipei City	298
Taichung City	175
**Tainan City**	**2853 (20.2%)**
**Kaohsiung City**	**8956 (63.9%)**
Keelung City	20
Hsinchu City	16
Chiayi City	13
New Taipei City	305
Taoyuan City	207
Hsinchu County	38
Yilan County	21
Miaoli County	29
Changhua County	79
Nantou County	31
Yunlin County	34
Chiayi County	33
Pingtung County	856
Penghu County	13
Hualien County	16
Taitung County	15
Kinmen County	4
Lienchiang County	1
Nation-wide	14,013

Based on information from the Center for Disease Control (CDC), Taiwan

**Fig. 2:**
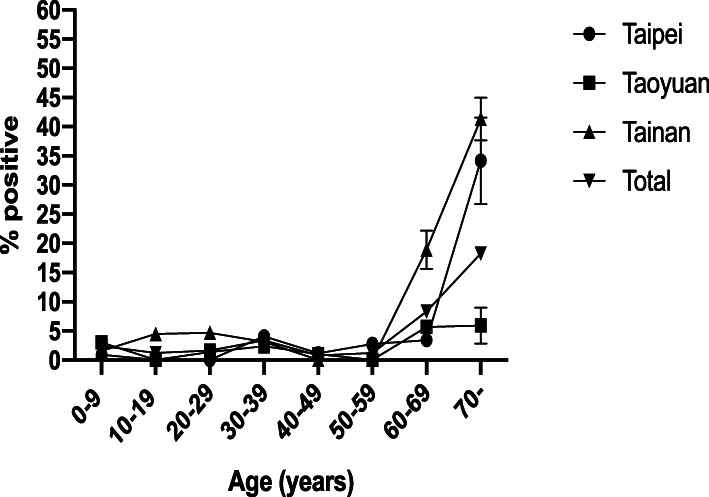
Age group-specific anti-DENV IgG seroprevalence by region

Figure 2 shows the prevalence of anti-dengue IgG in different age groups by regions. There was a significant association for increasing DENV IgG seropositivity with increasing age, similar to patterns in the overall group. A sharp rise in DENV IgG seropositivity in people 60 years and older was observed. Among those over 60 years of age, the highest seroprevalence was found in Tainan (41.31%), followed by that in Taipei (34.16%) and in Taoyuan (5.90%). As mentioned previously, there was a significant difference in the prevalence of DENV IgG among the 3 regions, and this difference was clearest in the age groups of 60 years and above.

## Discussion

Of the overall weighted prevalence of dengue-specific IgM and IgG antibodies of 6.78%, IgM was found positive in 1.76% and IgG in 3.40% of the study population. The seroprevalence of dengue antibodies in this study was lower than that reported from the epidemiological study conducted in nearby countries (39.3–59.0%) including Bangladesh, Singapore and India [19–21], but higher than that reported from Hong Kong (1.6%) [22]. Previous reports indicated that Taiwan is not a dengue endemic region and that the constant importation of DENVs from neighboring Southeast Asian countries is the major cause of dengue outbreaks; this may explain the relatively low seroprevalence of dengue-specific antibodies compared with the nearby endemic countries [6]. The discrepancy between DENV seroprevalence in Taiwan and that in Hong Kong may result from species of vectors. *Aedes aegypti*, the primary species responsible for transmitting dengue viruses, was not found in Hong Kong after the mid-1950s [22], and *Aedes albopictus* alone contributes to the transmission of dengue fever.

It has been wildly accepted that dengue epidemics transmitted by *Aedes albopictus* tend to be mild and short-lived. Correspondingly, the distribution of dengue cases highly overlaps where *Aedes aegypti* but not *Aedes albopictus* breeds in the field according to the survey conducted by Taiwan Centers for Disease Control (CDC). However, recent studies proposed that the propagation efficiency of *Aedes albopictus* for DENV1 and DENV3 transmission was as high a*s* that *of Aedes aegypti* and that their intimacy to people is the major reason why *Aedes aegypti* is the primary DENV vector. According to 2014 record-breaking dengue outbreak in Guangzhou and the ongoing locally transmitted dengue fever in Hong Kong, however, the role of *Aedes albopictus* in the transmission of dengue fever should not be neglected.

IgM- and IgG-capture ELISAs are widely used as diagnostic tests for confirmation of dengue virus infection and is considered to be a reliable serological test [23]. A patient who has IgM antibodies to dengue detected via ELISA indicates having a recent and primary dengue infection [24]. According to gender distribution, DENV IgM were more common in female subjects than males in this study, while no significant gender differences were observed for DENV IgG. These findings are comparable with the study done by Chien et al. [16].

In a serologic study of 1391 participants in in three administrative districts of Tainan in 2015, the seroprevalence of DENV IgM and DENV IgG were found to be increased with age [16]. In this study, however, there was no statistically significant difference in seroprevalence of DENV IgM among age groups, suggesting that age is not associated with the susceptibility to dengue fever. On the other hand, the multivariate analysis showed that older age was associated with DENV IgG seropositivity; most individuals less than 70 years old (born after 1940) were antibody negative (0–10%), and the positive rate reached up to 22.41% for those over 70 years old. This increase in seroprevalence is likely a reflection of the increasing exposure as individuals’ age. Moreover, the sharp increase in DENV seroprevalence in those aged 70 years and above could be partly attributed to the island-wide major outbreak of dengue in 1942, that resulted in 5 million infections among 6 million residents (> 80%) then [25]. The trends are in agreement with two other studies conducted in southern Taiwan (Tainan and Kaohsiung, respectively), in which drastic increase in seroprevalence of DENV IgG were found in both studies [16, 26, 27]. These findings also imply that the majority of population below 70 years of age are naive to all of the four dengue virus serotypes.

In the current study, region of residence was found to be significantly associated with the disparity of DENV IgG seropositivity. (Table 4) Individuals living in Tainan had 2.504 (95% CI = 1.262, 4.969) times the age-adjusted odds of being IgG seropositive compared with those living in Taipei, while those living in Taoyuan had 0.651 (95% CI = 0.275, 1.537) times the gender- and age-adjusted odds of being IgG seropositive compared with individuals living in Taipei. Considering the fact that the majority of outbreaks were reported from southern Taiwan, the discrepancy of seropositivity between Tainan and Taipei was relatively trivial. Population mixing facilitated by the efficiency of modern transportation networks may have accounted for that phenomenon. The relatively low dengue risk was observed in Taoyuan than that in Taipei may partially due to the lower population densities, lower people mixing with viremic individuals from southern Taiwan and lower people traveling abroad to dengue-endemic countries. Surprisingly, the seroprevalence of DENV IgMwas lowest in Tainan (1.50, 95% CI, 1.43–1.57%) and highest in Taoyuan (2.18, 95% CI, 2.12–2.25%), although the difference was not statistically significant. The similar probability of exposure may be explained by the fact that no dengue outbreak occurred in 2010. According to the reports from Taiwan Centers for Disease Control [28], a total of 1896 cases of dengue fever was reported in 2010 and 1106 cases resided in Kaohsiung City, accounting for 58.3% of the total cases. In the present study, the age -stratified seroprevalence of each city was investigated. As mentioned above, a sharp and significant increase in IgM seroprevalence from subjects aged below 70 years old to those ≥70 y/o was observed in Taipei and Taoyuan. On the contrary, the IgM seroprevalence was highest among individuals 0–9 years of age and no IgM seropositive case was detected in the 10–19-year-old, 50-year-old and over age group in Tainan. The absence of IgM antibodies in those age groups may be statistically error due to few dengue fever cases reported in 2010. Examining age patterns of DENV IgG seroprevalence, we found a sharp increase in DENV IgG seroprevalence for those aged 70 years and above presented in each city. These results confirmed the wide spread of dengue outbreak in 1942 or earlier. Moreover, the relatively low and stable rates among the age group of < 70 years indicated that the impact of outbreaks after1942 were relatively limited.

According to the multivariate analysis, immigrants were at significantly increased risk of DENV IgG seroprevalence. Unfortunately, the original nationality was not collected in the serosurvey in 2010. Further investigation is required to determine whether these individuals have acquired dengue infections before coming to live in Taiwan. However, most of recent immigrants were due to marriage and were from mainland China and Southeastern Asian countries where dengue is endemic.

According to the current study, there was only one sample positive for both IgM and IgG. This sample was collected from one 51-year-old male, who lived in Taipei with a household size of four. Unfortunately, his nationality was not documented then.

There were several limitations in this seroprevalence study. Firstly, the delinked serum samples were collected prior to the 2014–2015 outbreak of dengue fever in southern Taiwan, our results may not be representative to the current population profile. Second, some epidemiological factors that may influence the seropositivity were not collected. Traveling history, for instance, may impose significant effect on the seroprevalence since many endemic areas in Southeast Asia are popular destinations for Taiwan citizens. Moreover, sera samples from Kaohsiung region (in the southern part of Taiwan) were not included in this study. There was almost 40 years of dormancy after the island-wide dengue outbreak in 1942. However, the reemergence of dengue fever was documented in 1981 and dengue fever outbreaks occurred of different scales then. According to a database covering the period from 1998 to 2010 based on information from the Taiwanese Center for Disease Control (CDC) (Table 6), Kaohsiung accounted for 63.9% of 14,013 dengue cases reported in the entire country, responsible for most of the dengue cases in Taiwan. Therefore, the overall dengue disease prevalence in Taiwan in this study may be underestimated to some extent. A more recent and the inclusion of more districts are required for more precise conclusions about the association between risk factors and dengue seroprevalence in the current general population of Taiwan.

Conclusively, this study provides the baseline seroprevalence data of dengue fever in Taiwan, which included subjects from northern district and southern district of Taiwan. Further studies, which should include more districts of Taiwan and more sera samples obtained after 2014–2015 outbreak of dengue in Taiwan, are needed to realize the current situation of seroprevalence of dengue fever in Taiwan.

## Data Availability

The datasets used and/or analysed during the current study are available from the corresponding author on reasonable request.

## Abbreviations

DENV: Dengue virus
DSS: Dengue shock syndrome
DHF: Dengue hemorrhagic fever
ELISA: Enzyme-linked immunosorbent assay
